# 1. Chronic Gastritis. 2. Pernicious Anemia. 3. Distention of Stomach

**Published:** 1894-11

**Authors:** Francis Delafield

**Affiliations:** New York, Professor of the Practice of Medicine, College of Physicians and Surgeons


ATLANTA
MEDICAL AND SURGICAL JOURNAL.

VOL. XI. NOVEMBER, 1894. No. 9.

EDITED BY
LUTHER B. GRANDY, M.D., AND MILLER B. HUTCHINS, M.D.,
WITH THE CO-OPERATION OF
H. V. M. MILLER, M.D., LL.D., VIRGIL O. HARDON, M.D.,
FLOYD W. McRAE, M.D., DUNBAR ROY, M.D.,
AND H. R. SLACK, M.D., PH.M.

### *CLINICAL LECTURE.*

### 1. CHRONIC GASTRITIS. 2. PERNICIOUS ANEMIA. 3. DISTENTION OF STOMACH.

BY FRANCIS DELAFIELD, M.D., NEW YORK,
Professor of the Practice of Medicine, College of Physicians and Surgeons.

1. This man is fifty-five years old; a dyer by occupation. He has been a heavy drinker from time to time. He tells us that for the past ten months he has been losing flesh and strength, and according to his own account, this has not been accompanied by any impairment of his appetite. About five weeks ago, he began to experience a feeling of uneasiness in the epigastric region, and shortly after each meal, he had an attack of vomiting. He has not vomited either blood or mucus. These symptoms have continued up to the present time. His pulse to-day is 70; temperature, 98.4. The specific gravity of the urine is 1005; it contains a trace of albumin. The heart's action is regular; there is no murmur. The lungs give the physical signs of emphysema and bronchitis. The stomach is very much dilated. The amount of bronchitis he has would be sufficient to account for his loss of health during the past four years. He is also suffering from a chronic gastritis, probably due to alcohol, which has only recently given rise to pronounced symptoms. He may have a chronic gastritis which has gone on to stenosis of the pyloric orifice of the stomach, and which has produced a dilatation of the organ; or again, he may have a new growth at the pyloric end of the stomach, causing stenosis, and giving rise to the gastric symptoms. I think the diagnosis would lie between those three conditions. I doubt if it is possible to tell which of these conditions exists until the man has been under observation for some time. Taking all things into consideration, I should give the preference to the idea that he has a chronic gastritis—very likely alcoholic—with inflammatory stenosis of the pyloric end of the stomach. As regards treatment, I should recommend that the man be placed on a milk diet and have his stomach washed out—or be taught to do this himself—and that this be continued until he is able to retain solid food. Probably, after a few weeks of this treatment, one could be pretty certain which of these three conditions he has to deal with.

2. This patient is a man thirty-two years old. Twelve years ago he had typhoid fever, and was confined to the house for over two months. Since that time he has not been well. For several years he has suffered from malarial poisoning, having repeated attacks of intermittent fever. About four years ago he had an attack, during which he vomited blood, and blood was also passed in the stools. Last January he had a similar attack to this, vomiting blood and passing it from the bowels. Since then he has been very pale, as you see him now. During the past winter his weight has rathe increased than diminished, and he has gained in strength. Just now he has no very severe symptoms, excepting that he suffers from shortness of breath on exertion, and does not sleep well. His urine has a specific gravity of 1012; it contains a trace of albumin; no sugar. His temperature to-day is 99.6; pulse, 120. An examination of the chest shows hypertrophy of the left ventricle of the heart, and there is a systolic murmur heard in the second left intercostal space. There is no increased arterial tension. There is no enlargement of the liver or spleen. As you look at the man now, the thing most noticeable is his extreme pallor. Even without an examination of the blood, which is very important, but which has not yet been made, I think we would be fairly warranted in supposing that this man is suffering from one of two conditions, namely, (1) pernicious anemia, secondary to prolonged malarial poisoning, or (2) leucocythemia, without enlargement of the liver or spleen. The pernicious anemia is the more probable diagnosis, and for this we would put the man on arsenic and iron; the arsenic should be given in fair doses, say one-thirtieth of a grain of arsenious acid, or ten drops of Fowler's solution, three times daily. The iron could be given in the shape of Blaud's pills, two or three after each meal.

3. This man is thirty years old; a painter by occupation. Last Christmas he had an attack of illness which lasted for a couple of weeks, and shortly afterwards he was operated on for stricture of the urethra. With these exceptions, his health has apparently been good. Yesterday, while sitting quietly in the railway station, he suddenly felt as though he was about to lose consciousness. He was dizzy, and his heart seemed to stop beating. He did not actually lose consciousness. The attack lasted about ten minutes. Since then he has felt well. A physical examination of the lungs and heart gives negative results. There is no thickening of the arteries; no increased arterial tension. The point of importance in connection with this case is, what was the character of the attack which the man had yesterday? The diagnosis would lie between two conditions which have nothing to do with one another, and the treatment for which would be entirely different. The question is whether it was an actual attack of heart trouble, which we would class under the name of false angina, or whether the curious sensation experienced, namely, the vertigo, etc., were due to over-distention of the stomach. If we concluded that the attack was one of false angina, the treatment would consist in the use of potassium iodide. If, on the other hand, we come to the conclusion that the attack was due to the pressure of the distended stomach upon the heart, which I believe to be the case, we would ask the man to give up smoking and drinking alcoholic liquors and tea. For medicine we would give him the following:

| | |
|---|---|
| Sulpho-carbolate of soda | ʒ iv. |
| Tincture nux vomica | ʒ ii. |
| Glycerine | ℥ ii. |
| Aquæ ad. q. s. | ℥ vi. |

DOSE.—One tablespoonful three times daily.

